# Tumor-Specific Reactive Oxygen Species Accelerators Improve Chimeric Antigen Receptor T Cell Therapy in B Cell Malignancies

**DOI:** 10.3390/ijms20102469

**Published:** 2019-05-18

**Authors:** Hyeon Joo Yoo, Yibin Liu, Lei Wang, Maria-Luisa Schubert, Jean-Marc Hoffmann, Sanmei Wang, Brigitte Neuber, Angela Hückelhoven-Krauss, Ulrike Gern, Anita Schmitt, Carsten Müller-Tidow, Peter Dreger, Andriy Mokhir, Michael Schmitt, Leopold Sellner

**Affiliations:** 1 Department of Internal Medicine V, Heidelberg University Hospital, 69120 Heidelberg, Germany; hyeonjoo_7@hotmail.com (H.J.Y.); Yibin.Liu@med.uni-heidelberg.de (Y.L.); Lei.Wang@med.uni-heidelberg.de (L.W.); Maria-Luisa.Schubert@med.uni-heidelberg.de (M.-L.S.); Jean-Marc.Hoffmann@med.uni-heidelberg.de (J.-M.H.); Sanmei.Wang@med.uni-heidelberg.de (S.W.); Brigitte.Neuber@med.uni-heidelberg.de (B.N.); Angela.Hueckelhoven-Krauss@med.uni-heidelberg.de (A.H.-K.); Ulrike.Gern@med.uni-heidelberg.de (U.G.); Anita.Schmitt@med.uni-heidelberg.de (A.S.); Carsten.Mueller-Tidow@med.uni-heidelberg.de (C.M.-T.); Peter.Dreger@med.uni-heidelberg.de (P.D.); michael.schmitt@med.uni-heidelberg.de (M.S.); 2 National Center for Tumor Diseases (NCT), German Cancer Consortium (DKTK), 69120 Heidelberg, Germany; 3 Organic Chemistry II, Department Chemistry and Pharmacy, Friedrich-Alexander-University of Erlangen-Nürnberg, 91058 Erlangen, Germany; andriy.mokhir@fau.de

**Keywords:** chimeric antigen receptor, CAR, CART, immunotherapy, reactive oxygen species, ROS, chronic lymphocytic leukemia, CLL, aminoferrocene

## Abstract

Chimeric antigen receptor T cell (CART) therapy is currently one of the most promising treatment approaches in cancer immunotherapy. However, the immunosuppressive nature of the tumor microenvironment, in particular increased reactive oxygen species (ROS) levels, provides considerable limitations. In this study, we aimed to exploit increased ROS levels in the tumor microenvironment with prodrugs of ROS accelerators, which are specifically activated in cancer cells. Upon activation, ROS accelerators induce further generation of ROS. This leads to an accumulation of ROS in tumor cells. We hypothesized that the latter cells will be more susceptible to CARTs. CD19-specific CARTs were generated with a CD19.CAR.CD28.CD137zeta third-generation retroviral vector. Cytotoxicity was determined by chromium-51 release assay. Influence of the ROS accelerators on viability and phenotype of CARTs was determined by flow cytometry. The combination of CARTs with the ROS accelerator PipFcB significantly increased their cytotoxicity in the Burkitt lymphoma cell lines Raji and Daudi, as well as primary chronic lymphocytic leukemia cells. Exposure of CARTs to PipFcB for 48 h did not influence T cell exhaustion, viability, or T cell subpopulations. In summary, the combination of CARTs with ROS accelerators may improve adoptive immunotherapy and help to overcome tumor microenvironment-mediated treatment resistance.

## 1. Introduction

CD19-specific chimeric antigen receptor T cell (CART) therapy is currently one of the most outstanding cellular immunotherapy approaches, achieving substantial clinical benefit for patients with relapsed B-cell malignancies [1]. However, despite remarkable results, primary and secondary resistances occur. Escape mechanisms include down-regulation of tumor antigens [2], alterations of target antigens by alternative splicing [3], activation of alternative signaling pathways, and overexpression of inhibitory signals (e.g., cytotoxic T-lymphocyte-associated antigen 4 (CTLA4) or programmed cell death-1 (PD1)) [4]. Enhancing efficacy of CART therapy using combination strategies may be necessary to overcome resistance and achieve long-lasting remissions.

Cancer therapy against only a single target may facilitate the development of resistances. The combination with targeted drugs or immunomodulatory compounds may be a promising approach to improve clinical efficacy of CARTs. Previous studies reported that the addition of ibrutinib or lenalidomide to CART cells can improve effector functions in vitro, as well as tumor control in vivo [5,6,7]. Therefore, CART combination therapy approaches are a major focus of research efforts and may overcome treatment resistance towards adoptive immunotherapy.

The tumor microenvironment plays an important role in immune escape and tumor cell survival [8,9]. Therefore, it is of great interest to develop strategies to exploit these cancer-specific alterations by targeted combination therapies [10]. Metabolic alterations such as enhanced levels of oxidative stress and reactive oxygen species (ROS) are hallmarks of cancer [11]. Increased ROS levels have been demonstrated to be responsible for immune escape of cancer cells [12,13]. This crucial aspect of ROS-enrichment in the tumor microenvironment was exploited to design specific ROS accelerators for cancer treatment. They are activated in the presence of high ROS levels, and subsequently trigger further ROS production. This results in specific cell death of cancer cells [14,15,16]. Several such prodrugs have been reported. Organochalcogenide-based prodrugs can catalyze the oxidation of glutathione (GSH) to glutathione disulfide (GSSG) in the presence of H_2_O_2_ [17]. Furthermore, N-alkylaminoferrocene-based prodrugs are converted into ROS amplifiers (electron rich ferrocene) and alkylating agents (quinone methide). Both metabolites induce oxidative stress and lead to cancer cell death [14,15,16]. In previous studies, *N*-alkylaminoferrocene-based prodrugs demonstrated selective killing of primary chronic lymphocytic leukemia (CLL) cells, compared to healthy peripheral blood mononuclear cells (PBMCs) [14,15,16].

In this study, we focused on the N-alkylaminoferrocene-based ROS accelerator *N*-(3-(piperidin-1-ylmethyl)benzyl)-4-(ferrocenylcarbamatmethyl)phenyl boronic acid pinacol ester (PipFcB) as a tumor-specific anticancer agent with synergistic effects, in combination with CARTs. PipFcB is an experimental compound that is currently in preclinical evaluation [15]. Synergistic effects of PipFcB in combination with CD19-specific CARTs were observed by chromium-51 (^51^Cr) release assay in vitro. Furthermore, induction of intracellular oxidative stress by specific ROS accelerators was identified as a potential mechanism of these synergisms. 

## 2. Results

### 2.1. The ROS Accelerator PipFcB Increases CART-Mediated Lysis in Lymphoma Cell Lines

**Figure 1:**
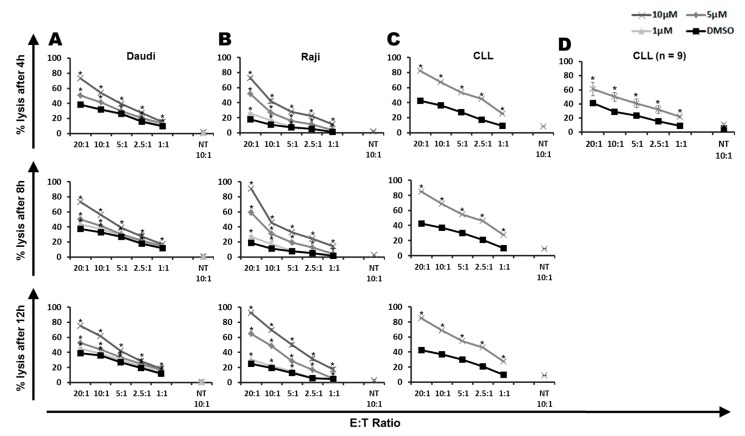
Influence of PipFcB on the cytotoxic capacity of chimeric antigen receptor T cells (CARTs) against Burkitt lymphoma lines and primary chronic lymphocytic leukemia (CLL) cells. Cytotoxicity of CD19-specific CARTs was determined by ^51^Cr release assay after co-culture with the CD19+ Burkitt lymphoma cell lines Daudi (**A**) and Raji (**B**), as well as primary CLL cells (**C**). Co-incubation with CART cells in different effector to target ratios (20:1, 10:1, 5:1, 2.5:1, 1:1) and non-transduced T cells (NT) was performed for 4 h, 8 h, and 12 h. Different concentrations of the specific reactive oxygen species (ROS) accelerator PipFcB (10 µM, 5 µM, 1 µM) or dimethyl sulfoxide (DMSO; vehicle) were added simultaneously with CARTs to the culture. Synergistic effects of CARTs with PipFcB were seen in all concentrations (1–10µM) and incubation times (4–12 h). Evaluation of primary CLL cells from nine different patient samples validated the synergistic effects of the combination of CARTs with PipFcB in primary leukemia cells (**D**). All experiments were performed in triplicates. Primary CLL cells were evaluated in nine independent experiments. Mean values were calculated for each group; error bars indicate standard deviation (* *p* < 0.05).

**Figure 2:**
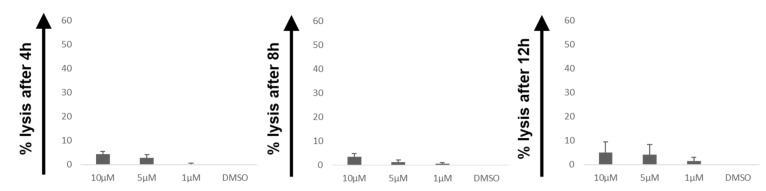
Direct lysis of Daudi cells by PipFcB. Cytotoxicity of PipFcB alone without CARTs was determined by ^51^Cr release assay after co-culture with Daudi cells for 4 h, 8 h, and 12 h. Different concentrations of the specific ROS accelerator PipFcB (10 µM, 5 µM, 1 µM) or DMSO (vehicle) were used. PipFcB as a monotherapy achieved only minimal lysis in the evaluated incubation times. All experiments were performed in triplicates and in three independent experiments. Mean values were calculated for each group; error bars indicate standard deviation.

The improved cytotoxic capacity of CARTs, in combination with the ROS accelerator PipFcB, was investigated at different incubation times (4, 8, and 12 h) in lymphoma cell lines. The combination showed significantly superior lysis compared to the dimethyl sulfoxide (DMSO) vehicle control in the CD19^+^ Burkitt lymphoma cell lines Daudi (Figure 1A) and Raji (Figure 1B) at all evaluated incubation times. The synergistic effect by PipFcB increased in a dose-dependent manner. Highest levels of lysis of the tested time points was achieved after 12 h incubation, at an effector:target (E:T) ratio of 20:1 and 10 µM PipFcB (PipFcB 10 µM vs. DMSO: Daudi 75% ± 2% vs. 39% ± 1%, *p* < 0.001, Raji 92% ± 1% vs. 25% ± 1%, *p* < 0.001). PipFcB alone, without CARTs, showed only minimal lysis in the evaluated concentrations and incubation times in Daudi cells (10 µM PipFcB: 5% ± 2%; Figure 2). The direct lysis of tumor cells by PipFcB cannot solely explain this major increase of lysis when combined with CARTs. 

### 2.2. The ROS Accelerator PipFcB Increases CART-Mediated Lysis in Primary CLL Cells

The improved cytotoxic capacity of CARTs, in combination with 10 µM of the ROS accelerator PipFcB, was investigated at different incubation times (4, 8, and 12 h) in primary CLL cells. The combination showed significantly superior lysis compared to the DMSO vehicle control in CD19^+^ primary CLL cells in all evaluated incubation times (Figure 1C). Highest increase of lysis was achieved after 12 h incubation at an E:T ratio of 20:1 (PipFcB 10 µM vs. DMSO: 87% ± 1% vs. 47% ± 1%, *p* < 0.001). This synergistic effect was reproducible in primary CLL cells from nine different patients (PipFcB 10 µM vs. DMSO: 67% ± 10% vs. 40% ± 2%, *p* < 0.001; Figure 1D). 

### 2.3. Pretreatment with the ROS Accelerator PipFcB Sensitizes Lymphoma Cells to CART-Mediated Lysis

**Figure 3:**
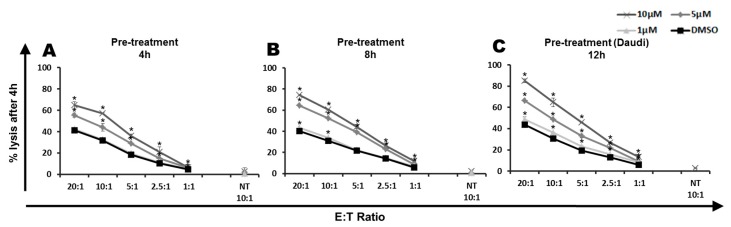
Pretreatment of lymphoma cells with PipFcB sensitizes lymphoma cells to lysis by CARTs. CD19^+^ Daudi cells were first incubated with different concentrations of PipFcB (10, 5, and 1 µM) or DMSO (vehicle) for 4 h (**A**), 8 h (**B**), or 12 h (**C**). Afterwards, CD19-specific CARTs in different effector to target (E:T) ratios (20:1, 10:1, 5:1, 2.5:1, 1:1) or non-transduced T cells (NT) were added and co-incubated with tumor cells for 4 h. Cytotoxicity was determined by ^51^Cr release assay. Synergistic effects of CARTs with PipFcB were seen in all concentrations (1–10 µM) and pre-treatment durations (4–12 h). All experiments were performed in triplicates. Mean values were calculated for each group; error bars indicate standard deviation (* *p* < 0.05).

To investigate if pretreatment of leukemia cells with PipFcB may sensitize to CART-mediated lysis, CD19^+^ Daudi cells were incubated for 4 h, 8 h, or 12 h with different concentrations of PipFcB (10, 5 and 1 µM), and afterwards exposed to CARTs at different E:T ratios (20:1, 10:1, 5:1, 2.5:1, 1:1) for 4 h (Figure 3). Pretreatment for 4 h significantly increased lysis with 10 µM and 5 µM PipFcB, compared to the DMSO control (E:T 10:1: 57% ± 1% and 44% ± 4% vs. 32% ± 1%, *p* < 0.001 and *p* = 0.004; Figure 3A). After 12 h pretreatment, significantly higher lysis was observed, even with 1µM PipFcB compared to the DMSO control (E:T 10:1: 36% ± 1% vs. 31% ± 1%, *p* < 0.001; Figure 3C). 

### 2.4. Increased Tumor Lysis by Combination of Other ROS Accelerators with CARTs

**Figure 4:**
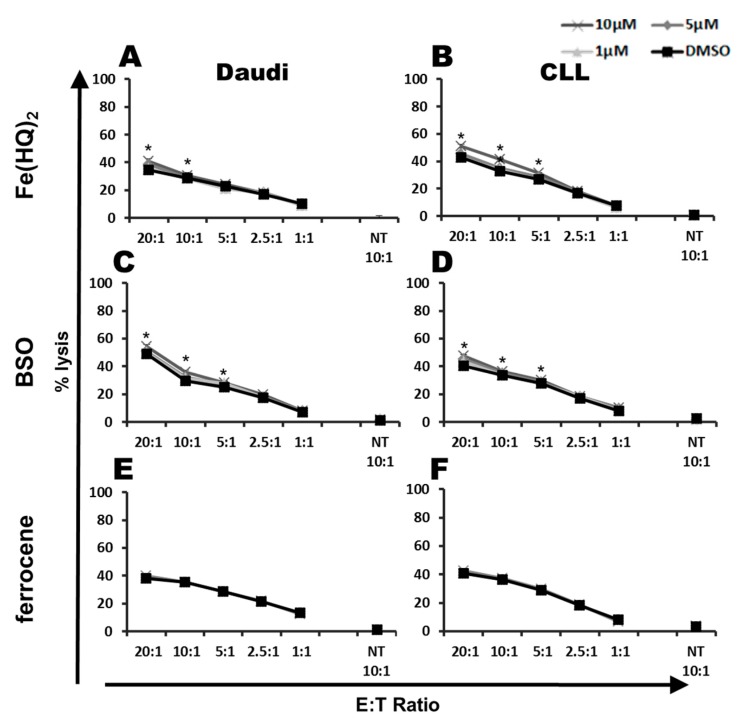
Influence of unspecific ROS accelerators and ferrocene on the cytotoxic capacity of CARTs. Cytotoxicity of CD19-specific CARTs was determined by ^51^Cr release assay after co-culture with the CD19^+^ Burkitt lymphoma cell line Daudi (**A**,**C**,**E**), as well as CD19^+^ primary CLL cells (**B**,**D**,**F**). Co-incubation with CARTs in different E:T ratios (20:1, 10:1, 5:1, 2.5:1, 1:1) and non-transduced T cells (NT) was performed for 4 h. Different concentrations of the unspecific ROS accelerators (10 µM, 5 µM, 2.5 µM) or DMSO (vehicle) were added simultaneously with CARTs to the culture. The unspecific ROS accelerators Fe(HQ)_2_ (**A**,**B**) and Buthionine sulfoximine (BSO; **C**,**D**) slightly increased the cytotoxic capacity of CARTs, but to a significantly lesser extent compared to specific ROS accelerators such as PipFcB. Ferrocene, the base compound of PipFcB without the capacity of ROS generation, did not influence specific tumor lysis by CARTs. All experiments were performed in triplicates. Mean values were calculated for each group; error bars indicate standard deviation (* *p* < 0.05).

To investigate if drug-mediated ROS acceleration is responsible for the synergistic effect of PipFcB, the combination of the two unspecific ROS accelerators—Fe(HQ)_2_ and Buthionine sulfoximine (BSO)—with CD19-specific CARTs was evaluated on CD19^+^ Daudi and primary CLL cells. The capacity for specific lysis of the weaker ROS accelerators Fe(HQ)_2_ (Fe(HQ)_2_ 10µM vs. DMSO, E:T 20:1: Daudi 41% ± 2% vs. 35% ± 2%, *p* < 0.001, CLL 51% ± 2% vs. 43% ± 1%, *p* < 0.001) and BSO (BSO 10 µM vs. DMSO, E:T 20:1: Daudi 54% ± 1% vs. 49% ± 1%, *p* < 0.001, CLL 47% ± 1% vs. 40% ± 1%, *p* < 0.001) combined with CARTs was significantly higher compared to the DMSO vehicle control (Figure 4A–D). However, the additional effect was clearly lower compared to the specific ROS accelerator PipFcB. A lower capacity of ROS-generation by Fe(HQ)_2_ and BSO may be responsible for these differences. To exclude other mechanisms than ROS-generation alone for synergistic lysis of aminoferrocene-based prodrugs when combined with CARTs, the effect of ferrocene, the base compound of PipFcB, in combination with CARTs was evaluated. Ferrocene has a high structural similarity to PipFcB but lacks the capacity of ROS-generation. This combination did not influence specific lysis of CARTs (Figure 4E–F). 

### 2.5. Influence of PipFcB on Lysis by CARTs in CD19^−^ Cells

**Figure 5:**
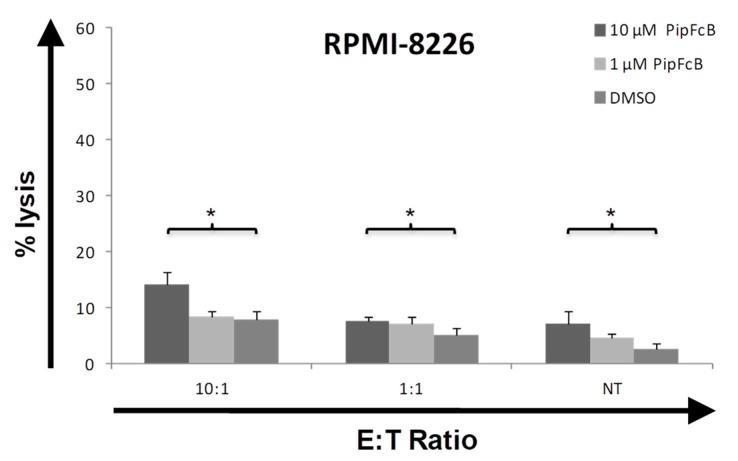
Influence of PipFcB on the cytotoxic capacity of CARTs against CD19^−^ cells. Cytotoxicity of CD19-specific CARTs was determined by ^51^Cr release assay after co-culture with the CD19^−^ multiple myeloma cell line RPMI-8226. Co-incubation with CARTs in different E:T ratios (10:1, 1:1) and non-transduced T cells (NT) was performed for 4 h. Different concentrations of the specific ROS accelerator PipFcB (10 µM, 1 µM) or DMSO (vehicle) were added simultaneously with CARTs to the culture. PipFcB significantly increased unspecific lysis by CD19-specific CARTs and NTs in CD19^−^ multiple myeloma cells. All experiments were performed in triplicates. Mean values were calculated for each group; error bars indicate standard deviation (* *p* < 0.05).

To exclude excessive increase of unspecific lysis by CD19-specific CARTs in the presence of PipFcB, cytotoxicity against CD19^−^ multiple myeloma cells (RPMI-8226) was evaluated. PipFcB significantly increased unspecific lysis by PipFcB compared to the DMSO control (10 µM PipFcB, E:T 10:1: 14% ± 2% vs. 8% ± 1%, *p* < 0.001; Figure 5), however, to a clearly lesser extent compared to effects on CD19^+^ cells.

### 2.6. No Influence of PipFcB on Degranulation or Intracellular Cytokine Production of CARTs

**Figure 6:**
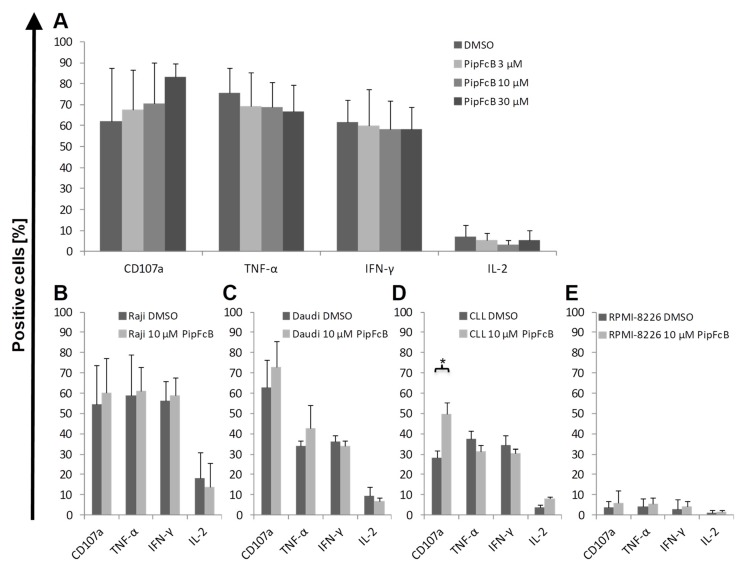
Influence of PipFcB on CART degranulation and intracellular cytokine production. CARTs were co-cultured for 6 h with Raji cells in the presence of PipFcB in different concentrations (3 µm, 10 µm, 30 µM). DMSO served as solvent control (**A**). Experiments were performed in three independent experiments. No differences in CD107a expression and intracellular production of TNF-α, IFN-γ and IL-2 was observed in the cultures supplemented with PipFcB, compared to the solvent control. 10 µM of PipFcB was further evaluated with CART stimulation by Raji (*n* = 6; **B**), Daudi (*n* = 3; **C**), primary CLL (*n* = 3; **D**), and RPMI-8226 cells (*n* = 3; **E**). No differences in CD107a expression and intracellular production of TNF-α, IFN-γ and IL-2 were observed in Raji-, Daudi-, or RPMI-8226-stimulated CART cultures supplemented with PipFcB, compared to the solvent control. The only significant difference by PipFcB was observed for CD107a expression on CARTs stimulated with primary CLL cells. Mean values were calculated for each group; error bars indicate standard deviation (* *p* < 0.05).

To determine if degranulation or cytokine production is influenced by ROS acceleration, CD107a expression and intracellular cytokine production of tumor necrosis factor-alpha (TNF-α), interferon-gamma (IFN-γ) and interleukin-2 (IL-2) was determined in the presence or absence of PipFcB. No induction of CD107a expression or intracellular cytokine production by PipFcB could be observed in CARTs after stimulation with the CD19^+^ Raji and Daudi cells (Figure 6A–C). The only significant difference by 10 mM PipFcB compared to the solvent control was observed for CD107a expression on CARTs stimulated with primary CLL cells (PipFcB 10 µM vs. DMSO 50% ± 6% vs. 29% ± 3%, *p* < 0.01; Figure 6D). No relevant changes in CD107a expression or cytokine production were observed by stimulation with CD19^−^ RPMI-8226 cells in the presence or absence of PipFcB (Figure 6E).

### 2.7. Malignant Lymphoma Cells are More Susceptible to PipFcB-Mediated Oxidative Stress 

**Figure 7:**
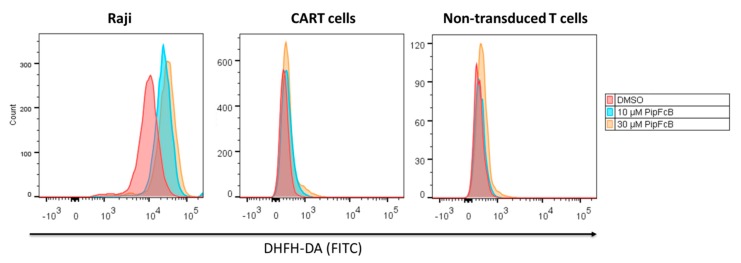
Generation of oxidative stress by PipFcB in malignant lymphoma cells, as well as CARTs and non-transduced T cells. After staining with Dichloro-dihydro-fluorescein diacetate (DCFH-DA), Raji cells—as well as CARTs and non-transduced T cells—were incubated with 10 µM or 30 µM PipFcB for four hours. DMSO served as solvent control. Raji cells exhibited a significantly higher induction of PipFcB-mediated oxidative stress. Representative histograms of three independent experiments are shown.

The generation of intracellular oxidative stress by PipFcB in tumor cells, as well as CARTs and non-transduced T cells, was investigated. Dichloro-dihydro-fluorescein diacetate (DCFH-DA) is a cell-permeable non-fluorescent probe that is oxidized to fluorescent 2′,7′-dichlorofluorescein in the presence of ROS. This effect can be utilized as an indicator for intracellular oxidative stress. Raji cells were significantly more susceptible to PipFcB-mediated induction of oxidative stress, compared to CARTs as well as non-transduced T cells (Figure 7).

### 2.8. Influence of Long-Term PipFcB Exposure on CARTs

**Figure 8:**
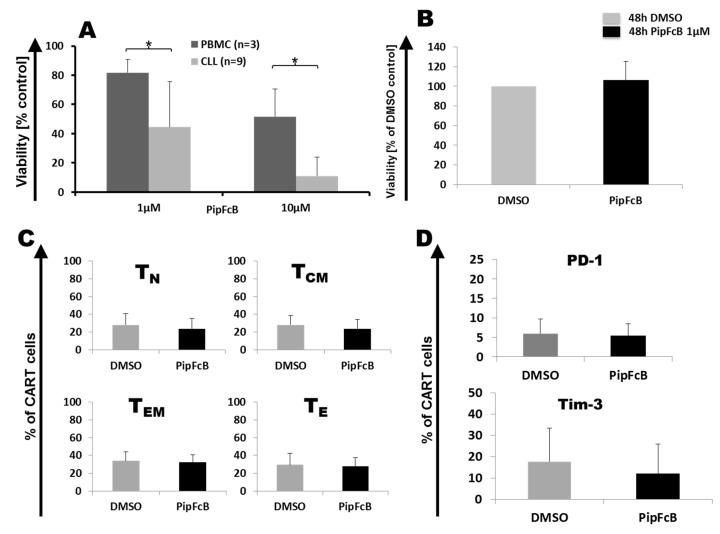
Long-term effects of the ROS accelerator PipFcB on primary CLL cells, as well as peripheral blood mononuclear cells (PBMCs) and CARTs. Healthy donor PBMCs (*n* = 3) and primary CLL cells (*n* = 9) were incubated with 10 µM and 1 µM PipFcB for 48 h. Viability was determined by quantification of ATP. 10 µM and 1 µM of PipFcB specifically killed CLL cells (**A**). However, 10 µM of PipFcB significantly reduced the viability of PBMCs. Therefore, 1 µM PipFcB was used to study long-term effects on survival and phenotype of CARTs. 1 µM of PipFcB did not influence viability of CARTs determined by NearIR in flow cytometry after cultivation for 48 h (*n* = 12) (**B**). In addition, no relevant effect of 1 µM PipFcB on naïve-like (T_N_), central memory-like (T_CM_), effector-memory-like (T_EM_), or effector-like (T_E_) CARTs (*n* = 12) (**C**)—or on the expression of the exhaustion markers PD-1 and TIM-3 (*n* = 12) (**D**)—was observed. Mean values were calculated for each group; error bars indicate standard deviation (*p < 0.05).

In order to study long-term effects of PipFcB on survival and phenotype, CARTs from six healthy donors were exposed for 48 h to 1 µM of PipFcB, the lowest dose with synergistic effects and little toxicity to healthy lymphocytes. Treatment of PBMCs from healthy donors (*n* = 3) and primary CLL cells (*n* = 9) showed that 10 µM of PipFcB is more specific in killing primary CLL cells after exposure for 48 h, but can also harm healthy PBMCs (viability PBMC vs. CLL 52% ± 19% vs. 11% ± 13%, *p* = 0.047, Figure 8A). 1 µM of PipFcB also displayed specific activity against CLL cells, while sparing healthy PBMCs (viability PBMC vs. CLL 82% ± 9% vs. 45% ± 31%, *p* = 0.01; Figure 8A). In addition, it showed synergistic effects with CARTs in short-term ^51^Cr release assay (Figure 1). 

For long-term culture, CARTs were cultured in medium supplemented with 10% human serum to increase survival and mimic in vivo conditions more accurately. 1 µM of PipFcB did not influence viability of CARTs (*n* = 12) after cultivation for 48 h (Figure 8B). 

There was no relevant effect of 1 µM PipFcB on T cell subsets (*n* = 12; Figure 8C). In addition, expression of the exhaustion marker PD-1 was not affected by 1 µM PipFcB after 48 h of co-incubation (*n* = 12; Figure 8D). However, a trend towards lower T-cell immunoglobulin and mucin-domain containing-3 (TIM-3) expression in CARTs after exposure to PipFcB for 48 h was observed (*n* = 12; without vs with 1 µM PipFcB 17% ± 16% vs. 12% ± 14%, *p* = 0. 1; Figure 8D). 

## 3. Discussion

Despite encouraging results, resistance of tumor cells against CARTs occurs. To therapeutically exploit immunosuppressive influences of the tumor-microenvironment, we evaluated synergistic influences and functional effects of ROS accelerators in combination with CD19-specific CARTs. Potential synergistic effects of CARTs in combination with tumor-specific ROS accelerators were identified. These are likely to be related to the induction of ROS generation by ROS accelerators. Combination therapy approaches may be a valuable option to overcome treatment resistance against CARTs. 

Reports from CLL patients treated with CD19-specific CARTs have demonstrated very encouraging effects and have been shown to provide long-term remissions [18,19,20]. However, compared to patients treated for diffuse large B cell lymphoma [21,22] or acute lymphoblastic leukemia [23], response rates ranging between 30–70% seem to be lower in CLL patients [18,19,20]. Therefore, optimization of CART therapy may improve the outcome of those patients. The combination of CARTs with small molecules or monoclonal antibodies currently presents as a major focus in adoptive immunotherapy. The Bruton’s tyrosine kinase (BTK) inhibitor ibrutinib [6,24], or immunomodulatory drugs such as lenalidomide [5,7], have been suggested to boost CART activity. Consequently, clinical trials with a combinatorial approach have been initiated (NCT02640209, NCT03070327). 

For targeted cancer treatment, approaches exploiting specific alterations of malignant cells for effective treatment whilst displaying little off-target toxicity are highly desirable. Lymphoid malignancies are known to display increased ROS levels. CLL cells, especially, seem to be exceptionally sensitive to ROS-generating compounds [25]. The mitochondrial metabolism was identified as one of the main sources for intracellular ROS accumulation and might be responsible for disease progression [26]. Therefore, this metabolic alteration represents a hallmark of cancer, and ROS may be an optimal therapeutic target in CLL. Pathogen-primed neutrophils and monocytes/macrophages can have relatively high ROS levels. Therefore, immunosuppression may be a side effect of tumor-specific ROS accelerators such as PipFcB. However, no severe immunosuppression or side effects against heart, liver, or kidney were observed in previously performed in vivo experiments in mouse models [15]. 

The ROS accelerator PipFcB significantly increased CART-mediated lysis in lymphoma cell lines, as well as primary CLL cells. Importantly, direct PipFcB-mediated lysis of tumor cells alone cannot explain significantly increased lysis in combination therapy, suggesting synergistic activities beyond additive effects. In addition, pretreatment of lymphoma cells with ROS accelerators sensitized malignant cells to lysis by CARTs. This indicates that the main effect of ROS accelerators is located within the target cells. This observation is supported by the fact that PipFcB significantly increased intracellular oxidative stress in tumor cells, whilst it did not influence non-transduced T cells or CARTs. Furthermore, cytokine secretion of CARTs was not affected by ROS accelerators. The increased degranulation was only observed in CARTs stimulated with primary CLL cells. Further investigation of this finding may be of interest. The unspecific ROS accelerators, Fe(HQ)_2_ and BSO, were also able to achieve synergistic activities in combination with CARTs, even though their effect was significantly lower than that of the specific ROS accelerator PipFcB. In contrast, ferrocene, the base compound of PipFcB, showed no effect against tumor cells. Therefore, intracellular ROS generation in tumor cells is likely to be mainly responsible for synergistic activities between CARTs and PipFcB. 

In previous studies, we demonstrated that aminoferrocene-based ROS accelerators—including PipFcB—are specific for tumor cells, whilst displaying little toxicity towards healthy PBMCs [14,15,16]. In the current study we confirmed this finding, demonstrating that PipFcB is significantly more toxic towards primary CLL cells compared to healthy PBMCs. However, 10 µM of PipFcB still displayed relevant toxicity to PBMCs, as well as significantly increased unspecific lysis by CARTs in CD19^−^ cells—even though, to a clearly lesser extent compared to lysis of CD19^+^ cells. In contrast, 1 µM of PipFcB that also achieved synergistic effects when combined with CARTs had little effects on viability, T cell differentiation, and expression of exhaustion markers. Therefore, analysis upon lower concentrations may be more suitable for in vivo evaluation of PipFcB in combination with CARTs in the future. Anti-tumor activity of PipFcB in mouse models with little toxicity against healthy tissue was demonstrated previously [15]. Optimization of currently available ROS accelerators for increased tumor specificity may reduce off-target toxicity of these compounds. This could be, for example, achieved by the addition of additional tumor-specific activation triggers such as hypoxia [27].

A limitation of this study would be the fact that the evaluation of combination therapy was carried out only in artificial two-dimensional cell culture conditions, which do not reflect the hypoxic conditions in the bone marrow or in the lymph node in vivo. Additional investigations in more complex culture conditions better reflecting the three-dimensional structure of tumor cells in vivo, including the tumor microenvironment [28], may help to further elaborate the mechanisms of ROS accelerator-mediated synergisms with CARTs. Furthermore, evaluation of ROS accelerators in combination with CARTs in mouse models is necessary to prove in vivo efficacy of this combinatory treatment approach.

CARTs might encounter significantly higher ROS levels in the immunosuppressive tumor-microenvironment in lymph nodes or the bone marrow. This could lead to decreased CART-mediated anti-tumor activity. Protection of CARTs with implementation of catalase in the chimeric antigen receptor (CAR) construct that can provide additional anti-oxidative capacity for CARTs [29], for example, might be helpful to overcome this limitation. Furthermore, an increased anti-oxidative capacity of CARTs may reduce the toxicity of ROS accelerators. Combination of such fourth-generation CARTs with tumor-specific ROS accelerators or other drugs with synergistic activities are likely to represent the future of CART-based treatment approaches [30]. 

In conclusion, the combination of ROS accelerators with CARTs might be an option to exploit the immunosuppressive tumor-microenvironment for optimized CART therapy.

## 4. Materials and Methods

### 4.1. Cell Lines

CD19^+^ Burkitt lymphoma (Daudi, Raji) and CD19^−^ multiple myeloma (RPMI-8226) cell lines were obtained from DMSZ (German Collection of Microorganisms and Cell Cultures, Braunschweig, Germany) and cultured in RPMI 1640 supplemented with 10% fetal bovine serum (FBS) and 2 mM l-glutamine (all Thermo Fisher Scientific, Waltham, MA, USA) at 37 °C and 5% CO_2_.

### 4.2. Primary Cells

Peripheral blood (PB) samples were collected from healthy donors (blood bank Mannheim, DRK-Blutspendedienst Baden-Württemberg-Hessen, Heidelberg, Germany) and CLL patients (University Hospital Heidelberg, Heidelberg, Germany). PBMCs were purified by ficoll density gradient (Linaris, Dossenheim, Germany) and cryopreserved. Informed consent was obtained from all patients according to the Declaration of Helsinki, and approval for the study was obtained from the Ethics Committee of the University of Heidelberg (16.06.2016; S-254/2016). CLL cells were cultured in RPMI 1640 supplemented with 10% Human Serum (Sigma-Aldrich, St. Louis, MO, USA) and 2 mM l-glutamine (Thermo Fisher Scientific).

### 4.3. Retroviral Vector Production

CD19.CAR-CD28-CD137zeta third generation retroviral vector was produced by co-transfecting 293T cells with the specific retroviral vector plasmid (3.75 µg), PegPam3 plasmid containing gag-pol (3.75 µg) and RDF plasmid containing the envelope (2.5 µg). Plasmids were kindly provided by Professor Malcolm Brenner, Center for Cell and Gene Therapy, Baylor College of Medicine, Houston, TX, USA. 

### 4.4. CD19-CART Generation and Culture

CD19-CART cells were generated as described previously [31,32,33]. In brief, PBMCs were thawed, cultured in 50% RPMI 1640 (Thermo Fisher Scientific) and 50% Click’s Medium (EHAA) (Irvine Scientific, Santa Ana, CA, USA), with 10% FBS (Thermo Fisher Scientific) and 2 mM L-glutamine (Thermo Fisher Scientific), and activated in 24-well plates (Corning, Wiesbaden, Germany) coated with anti-CD3 (OKT3; Biozol, Eching, Germany) and anti-CD28 (Fresenius, Bad Homburg, Germany) antibodies. On day 2, culture medium was supplemented with 4.4 × 10^3^ U/mL IL-7 and 100 U/mL IL-15 (both R&D Systems, Minneapolis, MN, USA). On day 3, activated T cells were transduced with the CD19.CAR-CD28-CD137zeta third generation retroviral vector in 24-well plates (Corning, Corning, NY, USA) coated with retronectin (Takara Bio, Shiga, Japan). On day 14, CARTs were cryopreserved in FBS (Thermo Fisher Scientific) + 10% DMSO (Sigma-Aldrich).

### 4.5. Compounds

The specific ROS accelerator PipFcB was synthesized as described previously [15]. Ferrocene (Sigma-Aldrich), the basic substance of aminoferrocene-based prodrugs without the function of ROS generation, served as a negative control [16]. The unspecific ROS accelerators Buthionine sulfoximine (BSO) and Fe(HQ)_2_ (Sigma-Aldrich) were used as positive controls. Compounds were dissolved in DMSO at a final concentration of 10 mM and stored at −20 °C. 

### 4.6. Chromium-51 Release Assay

Cytotoxic function of CARTs was determined using ^51^Cr release assay. CD19^+^ target cells were labeled with ^51^Cr (Hartmann Analytic, Braunschweig, Germany) for 2 h, followed by co-incubation with CD19-specific CARTs in 96-well U-bottom microplates (Greiner Bio-One, Frickenhausen, Germany) for different incubation times (4, 8, and 12 h). Different effector to target (E:T) ratios (20:1, 10:1, 5:1, 2.5:1, 1:1, and non-transduced T cells 10:1) with or without ROS accelerators (1–10 µM) were used. Maximum ^51^Cr release was assessed by addition of 1% Triton X-100 (Sigma-Aldrich). Background release was measured in the absence of CART cells. Culture supernatant was collected and diluted with Ultima Gold liquid scintillation cocktail (PerkinElmer, Waltham, Ma, USA). ^51^Cr release was analyzed by a 1414 WinSpectral liquid scintillation counter (PerkinElmer). All experiments were performed in triplicates. Specific lysis of ^51^Cr release was calculated as following: ((^51^Cr release in the test well − background ^51^Cr release)/(maximum ^51^Cr release − background ^51^Cr release)) × 100.

### 4.7. Immunophenotyping and Intracellular Cytokine Staining

Flow cytometry was performed on an LSRII device (BD Biosciences, Franklin Lakes, NJ, USA) and analyzed using FlowJo software (FlowJo, Ashland, OR, USA). Dead cells were excluded with the LIVE/DEAD™ fixable near-infrared(-IR) dead cell stain kit (APC-Cy7; Thermo Fisher Scientific). The goat F(ab)2 anti-human IgG (H+L) PE antibody (Dianova, Hamburg, Germany) was used for CD19-specific CAR expression. The following antibodies were used for immunophenotyping of surface markers: CD45RA-FITC, CCR7-PE-Cy7, CD8-Pacific Blue, PD-1-Alexa Fluor 488, Tim-3-Brilliant Violet 421 (all Biolegend, San Diego, CA, USA), CCR7-PE-Cy7, CD4-Alexa Fluor 700 (all eBioscience, San Diego, CA, USA), and CD3-AmCyan (V500) (BD Biosciences). Naïve-like T cells (T_N_) were defined as CD45RA+CCR7+; central memory-like (T_CM_) as CD45RA-CCR7+; effector memory-like (T_EM_) as CD45RA-CCR7-; and effector-like T (T_E_) cells as CD45RA+CCR7- T cells. Fixation of the cells after staining was performed with Dulbecco′s phosphate buffered saline (PBS; Sigma-Aldrich) containing 1% paraformaldehyd (PFA; Morphisto, Frankfurt am Main, Germany) and 3 mM ethylenediaminetetraacetic acid (EDTA; Sigma-Aldrich). Intracellular cytokine staining was performed as described previously [31]. In brief, cells were co-cultured with CD19-positive Daudi cells for 6 h in 96-well U-bottom microplates (Greiner BioOne, Frickenhausen, Germany). Brefeldin A (Biolegend) was used for intracellular cytokine retention according to the manufacturer’s protocol (5 h incubation at 37 °C). Fixation and permeabilization was performed with fix/perm solution and permeabilization buffer of the FoxP3 staining buffer set (Miltenyi Biotec, Bergisch Gladbach, Germany). Anti-interferon (IFN)-γ-Alexa Fluor 488 (Biolegend) and anti-tumor necrosis factor (TNF)-α-BV421 (BD Biosciences) were used for cytokine staining. 

### 4.8. Evaluation of Intracellular Oxidative Stress

Cells were incubated for 5 min with 10 µM DCFH-DA at 37 °C. After washing with PBS, Raji cells—as well as CART cells and non-transduced T cells—were incubated with 10 or 30 µM PipFcB for 4 h at 37 °C. DMSO served as solvent control. Flow cytometry was performed on an LSRII device (BD Biosciences) after stained for CD3-Alexa Flour 700 (Biolegend), CD20-Brilliant Violet 510 (Biolegend), and F(ab)2 IgG-PE (Dianova). Dead cells were excluded with the LIVE/DEAD™ fixable near-IR dead cell stain kit (Thermo Fisher Scientific).

### 4.9. Statistical Analysis

Statistical analyses were performed using Excel 2007 (Microsoft, Redmond, WA, USA). *p*-values were calculated using the parametric two-tailed t-test. Tests for two samples with equal variance (Figure 8A) or paired samples (other figures) were used. *p*-values < 0.05 were considered statistically significant. Graphs and tables were designed by Excel (Microsoft) and Origin Software 2016 (OriginLab Corporation, Washington, MA, USA). If not mentioned otherwise, results are presented as average ± standard deviation (SD).

## Abbreviations

^51^Cr	Chromium-51
BSO	Buthionine sulfoximine
BTK	Bruton’s tyrosine kinase
CAR	Chimeric antigen receptor
CART	Chimeric antigen receptor T cell
CLL	Chronic lymphocytic leukemia
CTLA4	Cytotoxic T-lymphocyte-associated antigen 4
DCFH-DA	Dichloro-dihydro-fluorescein diacetate
DMSO	dimethyl sulfoxide
FBS	Fetal bovine serum
IFN-γ	Interferon-gamma
IL-2	Interleukin-2
NT	Non-transduced T cells
PB	Peripheral blood
PBMC	Peripheral blood mononuclear cells
PD-1	Programmed cell death-1
PipFcB	N-(3-(piperidin-1-ylmethyl)benzyl)-4- (ferrocenylcarbamatmethyl)phenyl boronic acid pinacol ester
ROS	Reactive oxygen species
T_CM_	Central memory-like T cell
T_E_	Effector-like T cell
T_EM_	Effector memory-like T cell
T_N_	Naïve-like T cell
TIM-3	T-cell immunoglobulin and mucin-domain containing-3
TNF-α	Tumor necrosis factor-alpha
